# Droplet digital PCR allows vector copy number assessment and monitoring of experimental CAR T cells in murine xenograft models or approved CD19 CAR T cell-treated patients

**DOI:** 10.1186/s12967-021-02925-z

**Published:** 2021-06-21

**Authors:** Rafik Haderbache, Walid Warda, Eric Hervouet, Mathieu Neto da Rocha, Rim Trad, Vincent Allain, Clementine Nicod, Catherine Thieblemeont, Nicolas Boissel, Pauline Varlet, Ibrahim Yakoub Agha, Lucie Bouquet, Melanie Guiot, Fabienne Venet, Pierre Sujobert, Xavier Roussel, Paul-Oliver Rouzaire, Denis Caillot, Olivier Casasnovas, Jean Christophe Bories, Emmanuel Bachy, Sophie Caillat-Zucman, Marina Deschamps, Christophe Ferrand

**Affiliations:** 1 INSERM UMR1098, Right, EFSBFC, UFC, Laboratoire de Thérapeutique Immuno-Moléculaire Et Cellulaire Des Cancers, 8 rue du Dr Jean François Xavier Girod, 25020 Besançon, France; 2 Hôpital Saint-Louis, Assistance Publique-Hôpitaux de Paris (AP-HP), Université de Paris, Service d’Immunologie, Paris, France; 3 Hôpital Saint-Louis, Assistance Publique-Hôpitaux de Paris (AP-HP), Université de Paris, Service Hématologie, Paris, France; 4 Immunology Lab – HLA, Lille, France; 5 CHU de Lille, Univ Lille, INSERM U1286, Infinite, Lille, France; 6 Hospices Civils de Lyon, Immunology Laboratory, Edouard Herriot Hospital, Lyon, France; 7 Hospices Civils de Lyon, Hôpital Lyon Sud, Service d’Hématologie Biologique, Lyon, France; 8 UFR de Pharmacie, EA CHELTER 7453, CHU de Clermont-Ferrand, Clermont-Ferrand, France; 9 Hematology Clinical Department, Mitterrand Hospital, Dijon, France; 10 INSERM, UMR 976, Institut de Recherche Saint-Louis, Université de Paris, Paris, France; 11 Hospices Civils de Lyon, Hospital Lyon Sud, Service d’Hématologie Clinique, Lyon, France

**Keywords:** Chimeric antigen receptor, Droplet digital PCR, IL-1RAP, Tisa-cel, Axi-cel, Monitoring

## Abstract

**Background:**

Genetically engineered chimeric antigen receptor (CAR) T lymphocytes are promising therapeutic tools for cancer. Four CAR T cell drugs, including tisagenlecleucel (tisa-cel) and axicabtagene-ciloleucel (axi-cel), all targeting CD19, are currently approved for treating B cell malignancies. Flow cytometry (FC) remains the standard for monitoring CAR T cells using a recombinant biotinylated target protein. Nevertheless, there is a need for additional tools, and the challenge is to develop an easy, relevant, highly sensitive, reproducible, and inexpensive detection method. Molecular tools can meet this need to specifically monitor long-term persistent CAR T cells.

**Methods:**

Based on 2 experimental CAR T cell constructs, IL-1RAP and CS1, we designed 2 quantitative digital droplet (ddPCR) PCR assays. By targeting the 4.1BB/CD3z (28BBz) or 28/CD3z (28z) junction area, we demonstrated that PCR assays can be applied to approved CD19 CAR T drugs. Both 28z and 28BBz ddPCR assays allow determination of the average vector copy number (VCN) per cell. We confirmed that the VCN is dependent on the multiplicity of infection and verified that the VCN of our experimental or GMP-like IL-1RAP CAR T cells met the requirement (< 5 VCN/cell) for delivery to the clinical department, similar to approved axi-cel or tisa-cel drugs.

**Results:**

28BBz and 28z ddPCR assays applied to 2 tumoral (acute myeloid leukemia (AML) or multiple myeloma (MM) xenograft humanized NSG mouse models allowed us to quantify the early expansion (up to day 30) of CAR T cells after injection. Interestingly, following initial expansion, when circulating CAR T cells were challenged with the tumor, we noted a second expansion phase. Investigation of the bone marrow, spleen and lung showed that CAR T cells disseminated more within these tissues in mice previously injected with leukemic cell lines. Finally, circulating CAR T cell ddPCR monitoring of R/R acute lymphoid leukemia or diffuse large B cell lymphoma (n = 10 for tisa-cel and n = 7 for axi-cel) patients treated with both approved CAR T cells allowed detection of early expansion, which was highly correlated with FC, as well as long-term persistence (up to 450 days), while FC failed to detect these events.

**Conclusion:**

Overall, we designed and validated 2 ddPCR assays allowing routine or preclinical monitoring of early- and long-term circulating approved or experimental CAR T cells, including our own IL-1RAP CAR T cells, which will be evaluated in an upcoming phase I clinical trial.

**Supplementary Information:**

The online version contains supplementary material available at 10.1186/s12967-021-02925-z.

## Background

It is well known that the immune system is a remarkable barrier against cancer. Improvement of the immune system by ex vivo genetic engineering of immunocompetent T cells to express a chimeric antigen receptor (CAR T cells) has shown impressive and unexpected results in clinical trials for hematologic malignancies [1]. Consequently, treatment of refractory/relapsed (R/R) acute lymphoblastic leukemia (ALL) [2], diffuse large B cell lymphoma (DLBCL), and chronic lymphoid leukemia (CLL) [3] patients has led to approval, by US and EU regulatory agencies, of four autologous CAR T drugs directed against CD19 (tisagenlecleucel/tisa-cel/Kymriah®, Novartis; axicabtagene ciloleucel/axi-cel/Yescarta®, Kite-Gilead; brexucabtagene autoleucel/brexu-cel/Tecartus, Kite-Gilead; and lisocabtagene maraleucel/liso-cel/Breyanzi®, Bristol Meyer Squibb) and one against BCMA (idecabtagene vicleucel/ide-cel/Abecma®, BMS) for multiple myeloma [4]. This success greatly increases the likelihood of developing adoptive immunotherapy for other hematologic malignancies, such as multiple myeloma (MM) [5] and acute myeloid leukemia (AML) [6], as well as for solid tumors [7].

Pivotal clinical trials and routine practice [8] have shown that the first 28 days of peak expansion and the long-term memory persistence of CAR T cells are key factors in the efficiency and clearance of tumor cells. Moreover, adverse events such as cytokine release syndrome (CRS) and immune effector cell-associated neurotoxicity syndrome (ICANS) are observed after this therapy [9].

While an increasing number of patients are now routinely treated with these two approved drugs and many experimental CAR T cells are in development, there is a need for tools for monitoring circulating gene-modified T cells and evaluating the safety of advanced therapy medical products (ATMPs) for quality control of good manufacturing production (GMP) delivery, such as vector copy number per cell or transduction efficiency evaluation.

Independent of allogenic CAR T monitoring that can be performed using chimerism analysis methods, there are several additional ways to detect CAR T cells via in vivo tracking using methods such as PET imaging [10]. Flow cytometry analysis can be performed after indirect cell staining using a biotinylated protein target recognized by the CAR itself or by using monoclonal antibodies, allowing detection of cell surface expression of alternative proteins (truncated ∆CD19 [11], truncated nerve growth factor receptor ∆NGFR [12], short peptide/suicide epitope RQR8 [13], and truncated epidermal growth factor receptor ∆EGFR [14]) or CAR-coupled tags (HA and HIS) from onboard sequences of the CAR constructs.

Flow cytometry analysis is easy and rapid to perform and allows subcellular analysis among CAR T cell populations, but it needs to be performed on fresh samples, and the lack of sensitivity may impair analysis of long-term low-level CAR T cell persistence. Taking advantage of the presence of chimeric fusion nucleotide sequences coding for the CAR, molecular quantitative real-time PCR (qPCR) may be an ideal tool for quantitative determination of rare events, as it is already used in minimal residual disease quantification in oncohematology [15] or CAR T cell monitoring in peripheral blood [16]. More recently, droplet digital PCR (ddPCR), based on the multiplication of partitioned independent PCRs and Poisson statistics [17], has emerged as an easy, robust and reproducible molecular tool that may replace classical qPCR. The advantages consist of easy analysis of the endpoint and direct absolute quantification without the need for reference and calibration target DNA standard curves.

Here, we designed two ddPCR assays that target the T cell activation domain fusion areas CD28/CD3z (28z) and 4.1BB/CD3z (BBz) for tisa-cel and Axi-Cel sequences, respectively, and are useful for our own third-generation experimental IL-1RAP CAR construct [11]. ddPCR was compared to cytometry and qPCR before being applied to our preclinical ATMP production process in two in vivo xenograft experimental murine models (MM and AML) and in samples from patients treated for R/R ALL or BLBCL at different French clinical centers.

## Materials and methods

### Cell lines, CAR constructs and patient samples

The CEMT (CRL-2265™), HEK293T (CRL-11268™), MM1S (GFP+, Luciferase+, CRL-2974™) and Monomac6 (Luciferase+) cell lines were purchased from the American Type Culture Collection (ATCC) and stored in our master cell bank. Experimental CAR T cells were generated from healthy donors as described previously.

The CAR constructs included both approved CD19 CAR constructs (tisa-cel and axi-cel). We also used two experimental anti-IL-1RAP-CAR and anti-CS1-CAR vectors that mimic the intracellular activation domains of both commercial CAR T drugs. Axi-cel and tisa-cel are usually detected by flow cytometry using a biotinylated recombinant CD19 protein target. Our 2 experimental vectors carry truncated CD19 (∆CD19) and c-Myc Tag sequences, allowing for flow cytometry (FC) detection.

Blood samples were collected from patients treated with axi-cel or tisa-cel. All subjects provided written consent.

### Lentiviral supernatant production and CAR T cell manufacturing

Lentiviral supernatant was produced after triple transfection of the HEK293T cell line. Experimental CAR T cells were produced from healthy donors as previously described [11]. Briefly, cells were transduced after an initial activation and CD3+ MACS-selection step using CD3/CD28 beads following an expansion period in the presence of IL-2. Preclinical-grade IL-1RAP CAR T cells were also produced from healthy donors in a Prodigy device (Miltenyi Biotech) according to the manufacturer’s protocol. Briefly, selected CD4+ and CD8+ cells were activated with CD3/CD28+ IL-7/IL-15 (MACS GMP T Cell TransAct, Miltenyi Biotech) before being transduced and expanded for 9 days in a closed tubing set and TexMACS medium (Miltenyi Biotech). In both cases, genetically modified T cells were purified to increase purity to > 99%.

### Flow cytometry analysis

CEM-CAR and serial dilutions within the wild-type CEMT cell line and experimental CAR T cells were analyzed by flow cytometry on a BD LSRFortessa™ X-20 cell analyzer after staining with either FITC-cMyc antibodies (Miltenyi Biotec™, Clone SH1-26E7.1.3) for CS1 CAR T cells (Myc-tagged CS1 scFV) or APC-CD19 antibodies (Miltenyi Biotec™, Clone 6D5) for IL-1RAP CAR T cells (truncated CD19 is expressed on the cell surface of gene-modified T cells). 7-AAD staining was also performed to evaluate viability.

For patient monitoring, following incubation of whole blood with an FcR blocking reagent (Miltenyi Biotec), cells were stained with biotinylated human CD19 and Fc tag protein (AcroBiosystems) followed by phycoerythrin-labeled streptavidin (BioLegend). Classical T cell surface markers, including CD3, were also targeted with directly conjugated monoclonal antibodies. After red cell lysis, samples were analyzed in a FACSCanto™ II flow cytometer using FACSDIVA™ 8.0 software (BD Biosciences). The percentage of CAR+ T cells among CD3+ lymphocytes and the absolute number of CAR-T cells (deduced from bead-based absolute enumeration of T cells (BD Biosciences)) were determined.

### Primer and probe design

We designed two different quantitative PCR assays, 28z matching axi-cel and the CS1 experimental CAR and 41BBz matching tisa-cel and the IL-1RAP experimental CAR, and a PCR assay targeting GAPDH as a housekeeping reference.

**Fig. 1:**
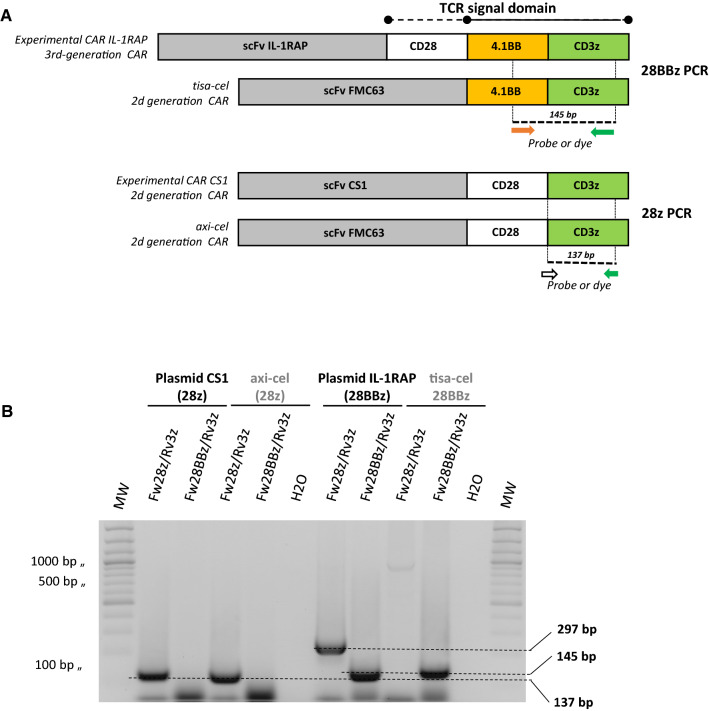
**A** Schematic representation of the CAR constructs and localization of PCR primers and probes. Localization of primers and probe for 28BBz and 28z PCRs specific for experimental IL-1RAP and tisa-cel CARs and for experimental CS1 and tisa-cel CARs, respectively. The sense primer for 28z PCR spans the CD28/CD3z junction area. FMC63: Single chain of the CD19 antibody. scFv: Single chain of the variable fragment. **B** Agarose gel electrophoresis of the 28z and 28BBz PCR products. MW: molecular weight marker; bp: base pair

Primers and probes were designed complementary to the sequences coding for the T activation domain of the CAR to match with the 2nd- or 3rd-generation CAR T constructs carrying the CD28 and 4.1BB genes. The CD28 forward primer used in 28z PCR spans the CD28/CD3z junction, and the bifluorescent TaqMan probe specific to CD3z is common for all CARs. The specificity of the primers was assessed by standard PCR performed with experimental CAR plasmid DNA or DNA extracted from left-over axi-cel or tisa-cel CAR T drug bags. For ddPCR, Evagreen (EvaGreen Supermix, Biorad, France) was used as a DNA-binding dye in place of the bifluorescent probe. A schematic representation and the sequences of primers and probes for PCR assays are provided in Fig. 1A and Table 1.

**Table 1:** Primer and probe sequences

Primers/probes		Name	Orientation	5′–3′ Sequence	Target area	PCR/Size of product (bp)	Targeted CAR/Genomic DNA
CAR	Primers	CD28j	Fw	CACGTCTCTTGTCCAAAACATC	Junction CD28/CD3z	28z/137	axi-cel; experimental CS1
		41BB	Fw	GAAGAAGAAGAAGGAGGATGTG	41BB	41BBz/145	tisa-cel; experimental IL-1RAP
		CD3z	R	CTTCGCAGCCTATCGCTCCAG	CD3z		Common to all CARs
	Probe [Fam/BHQ]	pCAR	Fw	CCCGCGTACCAGCAGGGCCAGA	CD3z		Common to all CARs
Reference	Primers	fGAPDH	Fw	ACATCATCCCTGCCTCTAC	GAPDH	GAPDH/179	Genomic DNA GAPDH specific
		rGAPDH	R	CTGCTTCACCACCTTCTTG			
	Probe [Hex/BHQ]	pGAPDH	Fw	CACTGCCAACGTGTCAGTGGTGGACCT	GAPDH		

### Extraction of genomic DNA from serial dilution of cells and plasmid DNA

Plasmid DNA was extracted using a plasmid DNA extraction kit (Qiagen, Courtaboeuf, France). Genomic DNA (gDNA) isolation was performed from cells or whole peripheral blood from patients using a QiaAmp Blood Extraction Kit (Qiagen, Courtaboeuf, France). DNA concentrations were measured by UV spectrophotometry using a Nanodrop ND2000 (Thermo Fisher, France), and the concentration was adjusted at 20 ng/μl. The absence of PCR inhibitors was checked by control PCR and agarose electrophoresis. Plasmid DNA was diluted into gDNA from the untransduced CEM cell line (from 10e6 to 10e0 copies). CS1- or IL-1RAP-transduced CEM cells were diluted into untransduced CEM cells and analyzed by flow cytometry. Moreover, gDNA was extracted from these 2 serial dilutions.

### qPCR and ddPCR amplification reactions

The qPCR mix was prepared by adding 10 µL of 2X TaqMan™ Universal Master Mix II buffer (Thermo Fisher, France), 1 µL of each primer at a concentration of 10 pmol/µL, 0.5 µL of probe at a concentration of 10 pmol/µL, 100 ng (equivalent to 150,000 cells) of DNA and H_2_O qs to 20 µL. The plate was centrifuged and loaded on a Bio-Rad™ CFX96 Real-Time PCR Detection System. The results were analyzed with the CFX Manager™ v3.1 Software.

Prior to ddPCR, DNA samples were either sonicated for 90 s (Covaris M220 ultrasonicator) or digested with the EcoRI enzyme, which is known to not cut within the amplification area. ddPCR mix was prepared using 10 µL of QX200™ ddPCR™ EvaGreen Supermix (BioRad, France), reverse and forward primers at final concentrations of 150 nM, 100 ng of gDNA (equivalent to 15,000 cells) and nuclease-free water in a total volume of 20 µL. The mixes were then loaded into the DG8™ Cartridge, and droplets were generated automatically with the QX200™ Droplet Generator. The emulsion was transferred to a PCR plate and cycled using the following thermal cycler conditions: predenaturation at 95 °C for 5 min, 40 cycles at 95 °C for 30 s, 60 °C for 1 min, and 4 °C for 5 min, and a final step at 90 °C for 10 min. Data acquisition and analysis were performed with the QX200™ Droplet Reader and QuantaSoft™ Software (Biorad, France).

### CAR copy quantification, vector copy number quantification and multiplicity of infection

Typically, CAR quantification is given as the number of transgene copies per µg of gDNA, adjusted from the starting quantity of gDNA used in PCR. In practice, while 100 ng of gDNA is equivalent to 15,000 cells or 30,000 haploid gene copies, it is possible to translate and thus estimate the number of cells, hypothesizing that there is one copy of vector per cell.

Vector copy number (VCN) was assessed by ddPCR after running both target and reference GAPDH PCR. Assuming that there were 2 copies of the GAPDH gene per genome cell, the vector copy number (VCN) was calculated from absolute ddPCR quantification as a ratio of the number of target copies to half the number of reference gene copies normalized by the transduction efficiency percentage.

We also investigated the link between the viral particle number (multiplicity of infection) used for transduction and the transduction efficiency and transgene copy number. Transductions were performed at different MOIs (0.02, 0.04, 0.1, 0.2, 1, 10, 25, and 50) for each CAR, as previously described. Transduction efficiencies were measured at day 9 prior to DNA extraction and ddPCR.

### CAR T cell monitoring in AML and MM xenograft tumor murine models

NOD/SCID/IL2Rγc-deficient (NSG) mice (6–8 weeks of age, The Jackson Laboratory, Sacramento, CA, USA) were irradiated (2.5 Gy) and inoculated intravenously (I.V.) with luciferase-expressing MM1-S/CS1+ (MM) or MonoMac-6/IL1RAP+ (AML) cell lines (1.10e6/mouse). Twenty hours later, the mice were infused I.V. with 5.10e6 CS1- or 10.10e6 IL-1RAP CAR T cells. For each model (n = 8 mice), the controls consisted of irradiated mice only (n = 1), mice infused with CAR T cells only (n = 3) or mice xenografted with tumor cell lines (n = 1). Tumor growth was studied by luminescence imaging (IVIS 152 Lumina Series III, Perkin Elmer, Waltham, MA, USA). Peripheral blood was harvested every 3 days by submandibular bleeding. At the end of the experiment (day 30), the mice were euthanized, and the bone marrow, spleen and lung were harvested for DNA isolation and ddPCR. All animal procedures were carried out according to the guidelines of our animal experimentation house. The area under the curve (AUC) was calculated from day 0 to day 28 using GraphPad Prism 8.0 software.

### Monitoring of patients treated with approved CAR T cell drugs

ddPCR assays were performed retrospectively or prospectively for peripheral blood monitoring of axi-cel- or tisa-cel-treated patients (B-ALL or DLBCL) from different clinical centers and compared with flow cytometry monitoring using biotinylated recombinant CD19 protein. CD19+ cell quantification was also performed by flow cytometry. Among 17 patients monitored over a period covering early extension (D0 to D28) and long-term persistence (up to D450), 10 patients were infused with axi-cel, and 7 were infused with tisa-cel. Patient characteristics and outcomes are provided in Table 2.

**Table 2:** Characteristics and outcomes of patients

ID	Age	Gender	Disease	CART inj	CART	CRS		ICANS		Day 30 PET-scan	Follow-up	Outcome (Feb 19, 2021)
						Grade	Post infusion	Grade	Post infusion			
UPN #1	65	F	NHL GCB 3rd line	January 27, 2020	axi-cel	2	D2	*NA*	*NA*	CR	CR (M12)	Alive
UPN #2	67	M	NHL GCB 3rd line	February 7, 2020		0	*NA*	*NA*	*NA*	CR	Relapse (M12)	Alive
UPN #3	66	M	NHL GCB 3rd line	February 21, 2020		2	D2	4	D5	CR	Relapse (M18)	Alive
UPN #4	72	F	NHL GCB 3rd line	May 12, 2020		2	D12	2	D17	CR	CR (M110)	Alive
UPN #5	67	M	NHL GCB 3rd line	May 25, 2020		2	D5	2	D11	CR	Relapse (M14)	Dead (Oct 3, 2020)
UPN #6	65	M	NHL GCB 4th line	June 2, 2020		2	D1	3	D6	CR	CR (M8)	Alive
UPN #7	52	M	NHL GCB 4th line	June 12, 2020		2	D2	1–2	D14	PR	CR (M8)	Alive
UPN #8	70	F	NHL GCB 3rd line	July 20, 2020		1	D2	3	D5	Progression	Refractory	Dead (Oct 8, 2020)
UPN #9	54	F	DLBCL (tFL), 3rd line	May 13, 2019		0	*NA*	0	*NA*	CR	CR (M12)	Alive (Jun 4, 2020)
UPN #10	69	F	DLBCL NGC, 5th line	July 11, 2019		2	D5	2	D6	CR	CR (M12)	Alive (Jul 9, 2020)
UPN #11	25	M	B-ALL, 3rd line	April 3, 2019	tisa-cel	0	*NA*	0	*NA*	CR, negative MRD	persisting B cell aplasia (M23), CD19low relapse (M23)	Alive (Mar 5, 2021)
UPN #12	55	M	DLBCL GC, 3rd line	July 9, 2019		2	D3	0	*NA*	PR	CR (M18)	Alive Jan 11, 2021
UPN #13	62	M	DLBCL (tFL), 6th line	June 4, 2019		0	*NA*	0	*NA*	CR	CR (M18)	Alive (Mar 1, 2021)
UPN #14	23	M	Phi + B-ALL, previous allograft, 2nd line	June 12, 2019		1	D8	0	*NA*	CR, negative MRD	Loss of B cell aplasia (M6), molecular relapse (M6), CR with negative MRD (M19) after TKI treatment	Alive (Jan 21, 2021)
UPN #15	56	M	DLBCL (tFL), 4th line	September 24, 2019		2	D4	1	D7	CR	CR (M17)	Alive (Feb 18, 2021)
UPN #16	15	M	B-ALL, previous allograft, 4th line	January 28, 2020		0	*NA*	0	*NA*	CR, negative MRD	CD19 + relapse (M6)	Alive (Feb 18, 2021)
UPN #17	19	F	B-ALL, 3rd line	June 16, 2020		0	*NA*	0	*NA*	CR, negative MRD	CR with negative MRD (M1), Loss of B cell aplasia (M2), CR (M2), follow up in another center	Alive (Aug 17, 2020)

## Results

### Quantitative PCR assays amplify both DNA from experimental and approved drugs

We first verified that our own PCR assays designed from the known sequences of the experimental CAR sequences matched and amplified sequences from genomic DNA extracted from axi-cel or tisa-cel CAR T drug bags. As reported in Fig. 1B, PCR products of the expected size were detected for the 28z plasmid and axi-cel (137 bp) or for the 28BBz plasmid and tisa-cel (145 bp). Fw28z/Rv3z PCR amplified both experimental plasmids (297 bp) but not tisa-cel DNA because the CD28 sequence was missing. To confirm this hypothesis, Sanger sequencing of the PCR products obtained after amplification from axi-cel and tisa-cel DNA showed 100% homology between all respective sequences (data not shown).

**Fig. 2:**
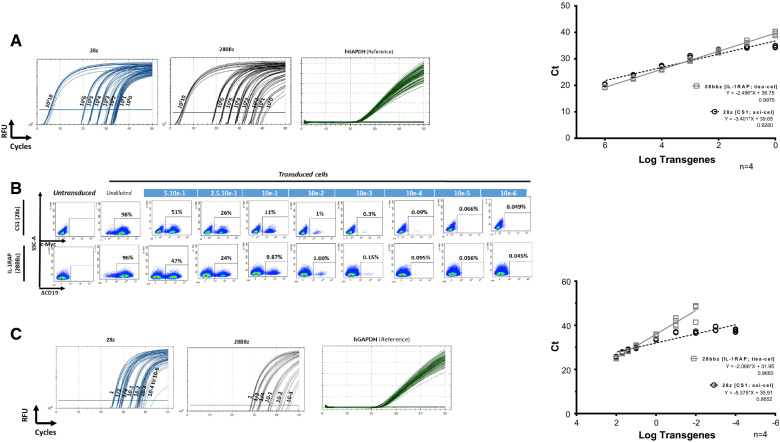
**A** Real time qPCR of DNA plasmids. 28z and 28BBz plasmids were serially diluted in untransduced CEM DNA and amplified by PCR with both Fw28z/Rv3z and GAPDH primer pairs. Amplification curves are provided (left). Ct is plotted against log transgene dilution, and linear regression equations and correlation coefficients are provided (right) for n = 4 independent experiments. **B** Flow cytometry-based CAR T cell detection. CS1- or IL-1RAP-transduced CEM cell lines were serially diluted in an untransduced CEM cell line. The percentage of the starting transduced cell population is provided. Representative experiment. SSC-A: side scatter. **C** Real-time qPCR of genomic DNA from serially diluted CAR-transduced cells. Genomic DNA was extracted from CS1- or IL-1RAP-transduced CEM cells serially diluted in untransduced CEM and amplified by 28z, 28BBz and GAPDH qPCR. Amplification curves are provided (left). Ct is plotted against log transgene dilution, and linear regression equations and correlation coefficients are provided (right) for n = 4 independent experiments. RFU: relative fluorescence units; Ct: cycle threshold; Ct: cycle threshold

The TaqMan qPCR method was first applied to the experimental plasmids IL-1RAP and CS1 serially diluted into human genomic DNA from healthy donors or to genomic DNA extracted from CEM gene-modified T cells diluted into normal CEM cells. As reported in Fig. 2A, B and Additional file 1: Table S1, both 28z and 28BBz PCR assays were able to detect one copy of plasmid in the starting nucleic material. Regression curves were linear for either plasmid or cell DNA dilutions, with correlation coefficients of R^2^ = 0.928 and 0.8652 or R^2^ = 0.9975 and 0.9683, respectively, for 28z or 28BBz PCR. Remarkably, the 28z PCR requires fewer amplification cycles (Ct) than the 28BBz PCR to detect one copy of plasmid (34.53 ± 0.34 and 39.68 ± 0.67, respectively).

### Quantitative ddPCR is more or more sensitive than flow cytometry

**Fig. 3:**
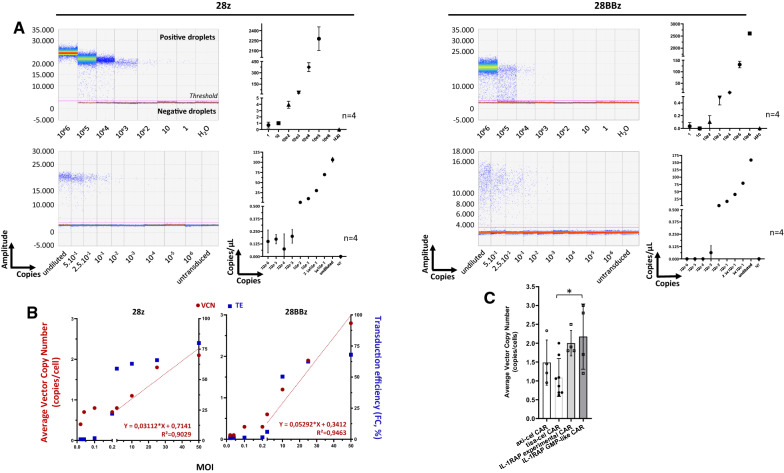
**A** ddPCR sensitivity assessment by plasmid dilutions. Both plasmids were diluted in nontransduced CEM cell gDNA, and CEM-CAR was diluted in an untransduced CEM cell line. ddPCR (28z and 28BBz) was applied to plasmid dilution (top) and to gDNA extracted from the cell line dilution (bottom). Positive and negative droplets were delimited by the threshold (pink line). The CAR copy number is plotted against plasmid copy numbers or cell dilution factors. Linear regression curves and correlation coefficients are provided for n = 4 independent experiments. CAR DNA plasmids (10e6 copies) or CAR + cell lines (undiluted) are considered as positive PCR controls. NT: not transduced sample. **B** Vector copy number. (vector copy number (VCN, transgene copies/CAR T cells, Y left axis, red circles) is plotted against multiplicity of infection (MOI) calculated from 28z and 28BBz ddPCR and reference housekeeping gene (GAPDH) ddPCR normalized to the transduction efficiency percentage. Transduction efficiency (TE, blue squares) obtained by flow cytometry (FC, %) is provided (Y right axis). (Left) Average VCN measured for axi-cel (n = 4) and tisa-cel (n = 8) obtained from leftover bags of CAR T cell products and for IL-1RAP CAR T cells produced under experimental (n = 4) or GMP-like conditions (n = 4). *p < 0.05

Validated qPCR assays were then switched to ddPCR assays and applied to the same plasmid DNA or gDNA cell dilutions. As reported Fig. 3A and Additional file 1: Table S2, 28z and 28BBz ddPCR are also able to detect copies of the target throughout the dilution range for plasmid DNA (up to one copy) but are only able to detect up to 10-e3 copies for gDNA cell dilutions, even if the target is always detectable (28z PCR). Flow cytometry detection (Fig. 2B, Additional file 1: Table S3) allowed us to detect precisely 10E−3 after either CS1 or CD19 staining, while ddPCR allowed detection at a level of 10e−3 (for 28 BBz) and 10E−6 (for 28z).

### Vector copy number, analyzed by ddPCR, is dependent on the MOI but meets the regulatory requirement for the “GMP-like” IL-1RAP CAR T cell preclinical production process

Mean vector copy number per cell was quantified with ddCPR targeting CAR T vector sequences and a GAPDH housekeeping gene to estimate the number of genome equivalents (assuming that there were 2 copies of the gene per cell). The results were normalized according to the percentage of gene-modified T cells obtained by flow cytometry (experimental or GMP-like IL-1RAP CAR, n = 4) or from the drug leaflet (n = 4 or 8, respectively, for axi-cel or tisa-cel) provided with the infusion bags. As reported in Fig. 3B, for an MOI < 1 [0.02–0.2], the average VCN per cell remained under 1 [0.4 to 0.8 and 0.1 to 0.6 for CS1 (28z) and IL-1RAP (28BBz) CAR, respectively]. However, when the MOI is ≥ 1 [1–50], the mean VCN per cell increased linearly [1.1 to 2.1 (R^2^ = 0.9) and 1.2 to 2.8 (R^2^ = 0.94) for CS1 (28z) and IL-1RAP (28BBz) CAR, respectively]. Accordingly, transduction efficiency, assessed by flow cytometry, correlated with VCN according to MOI.

We then quantified the average VCN for DNA extracted from leftover bags of axi-cel or tisa-cel samples (Table 3). The means VCN was 1.5 ± 0.6 (n = 4) and 0.9 ± 0.39 (n = 8) for transduction efficiencies of 70.3 ± 9.67% and 17.7 ± 11.74%, respectively. For IL-1RAP CAR T cell production, either experimental or GMP-like, we did not note a significant difference between the average VCN per cell of IL-1RAP CAR T cells, with values of 2 ± 0.34 (n = 4) and 2.2 ± 0.87 (n = 4) for transduction efficiencies of 27.4 ± 9.67% and 29.6 ± 17.50%, respectively (Fig. 3C).

**Table 3:** Transduction efficiency and VCN

CAR products			Transduction efficiency (%)	VCN
CART Bags Leftover	#1	tisa-cel	11.2	0.86
	#2		13.9	0.69
	#3		14.7	1.06
	#4		46	1.09
	#5		17.7	0.62
	#6		10.1	1.69
	#7		16.6	0.69
	#8		11.4	2.0
	#9	axi-cel	58	2.33
	#10		78	1.45
	#11		78	0.95
	#12		67	1.21
Local production (experimental)	#13	IL-1RAP	11.1	2.5
	#14		55.8	1.8
	#15		24.5	1.9
	#16		18	1.8
Local production (GMP-like)	#17	IL-1RAP	55.3	1.2
	#18		15.9	1.7
	#19		23.9	2.8
	#20		23.4	3.0

### ddPCR allows in vivo monitoring of experimental CAR T cells in mouse tumor xenograft models

**Fig. 4:**
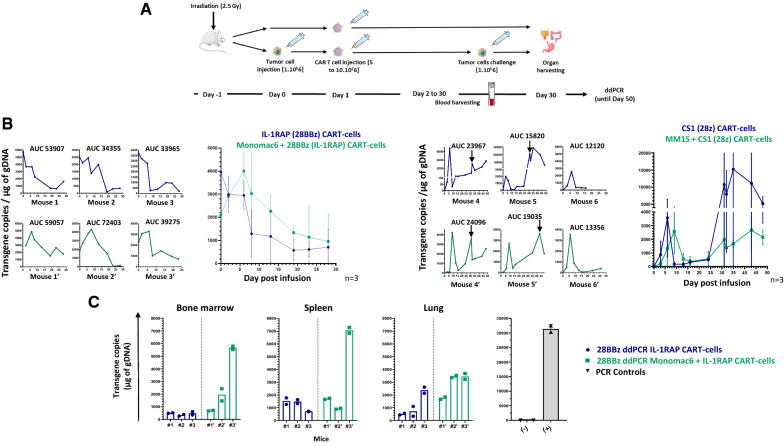
In vivo CAR T cell monitoring method for xenograft tumor murine models. **A** Schematic workflow summarizing the xenografting of MM1S or Monomac-6 tumor cell lines (day 0) one day after mouse irradiation (2.5 Gy). On day 1, CS1 or IL-1RAP CAR T cells were intravenously injected. Biological samples were harvested kinetically (PB, day 2 to day 30 or 50) or at the end of the experiment (organs, day 30, Monomac-6/IL-1RAP) and processed for gDNA extraction and ddPCR. For the MM1S/CS1 model, mice were challenged again with tumor cells; n = 3 mice (12 in total) were included in each group. **B** Individual early (day 0 to day 50) longitudinal monitoring of CS1 and IL-1RAP monitoring with 28z and 28BBz ddPCR, respectively. Green and blue curves represent ddPCR CAR T cell monitoring for mice grafted or not with the target tumors, respectively. The mean CAR transgene copy number detected in each group is plotted (n = 3 mice/group). Arrows show the second CAR T cell expansion peaks following the second tumor injection. AUC: area under the curve day 0 to 28, calculated with GraphPad software. **C** Monitoring CAR T cells in organs. Transgene copies/µg of DNA obtained by 28BBz ddPCR on gDNA extracted from organs of n = 3 individual mice receiving IL-1RAP CAR T cells after grafting (green) or not (blue) of Monomac6 tumor cells. Each quantification was performed in duplicate. (−) and (+) are negative and positive PCR controls as respectively no template or IL-1RAP CAR+ cell line DNA

We then applied our 28z and 28bbz ddPCR assays to peripheral blood harvested from mice (n = 3, in each group) xenografted or not with tumor cell lines (AML, Monomac-6/IL-1RAP CAR for 28BBz and MM, MM1S/CS1 CAR for 28z). In the MM1S/CS1 model, after early CAR T cell responses, the mice were challenged again with a new infusion of the tumor cell line. At the end of the experiment, after serial harvesting of PB, organs (marrow, spleen, and lungs) were collected (Fig. 4A). We demonstrated that our ddPCR assays are able to monitor the early expansion of circulating CAR T cells in both models and allow us to calculate the area under the curve (AUC_0-28_). For the AML model, we showed a difference in CAR T cell expansion in the presence (AUC_0-28_ = 56911 ± 16668, n = 3) and absence (AUC_0-28_ = 40452 ± 11403, n = 3) of target cells. For the MM model, we did not detect a significant difference between CAR T cell expansion in mice xenografted (mean AUC_0-28_ = 18895 ± 5373, n = 3) or not (mean AUC_0-28_ = 17302 ± 6061, n = 3) with tumor cells. However, in the MM model, when the mice were challenged again with the tumor, we clearly observed a second peak expansion in 4 out of 6 mice independent of whether the mice were grafted or not with tumor MM1S cells at day 0 (Fig. 4B).

Finally, we showed that the 28BBz ddPCR assay is useful for quantifying the homing of CAR T cells. As shown in Fig. 4C, IL-1RAP CAR T cells were found (on day 31) in all the investigated organs, such as the bone morrow (n = 3), spleen (n = 3) and lung (n = 3), with a trend of higher amounts in mice grafted with AML tumor cells.

### ddPCR allows patient monitoring of early increases and long-term circulation in the peripheral blood of both approved CD19 CAR T cells

**Fig. 5:**
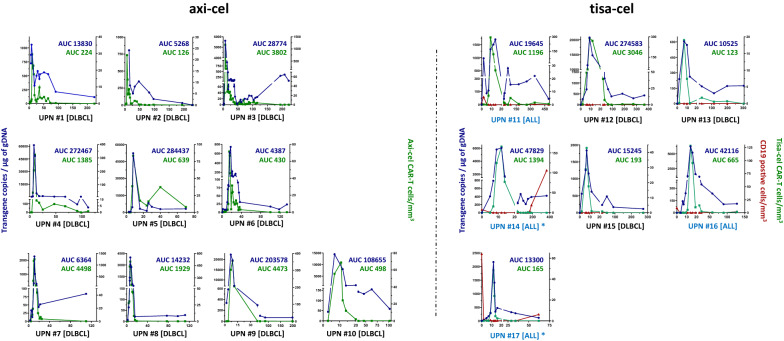
Long-term monitoring and follow-up of CAR T cells in treated DLBCL or B-ALL patients. Ten patients (DLBCL) receiving axi-cel and 7 (4 B-ALL and 3 DLBCL) receiving tisa-cel were monitored using 28BBz or 28z ddPCR (blue lines, left Y-axis) on gDNA extracted from circulating PB cells. CD19 CAR T cell numeration (red lines, right Y-axis), obtained by FC, is plotted (green lines, right Y-axis) on the same respective graph. AUC: area under the curve day 0 to 28, calculated with GraphPad software. (*) highlights patients in whom CD19+ cells reappear

We finally performed a prospective and retrospective study by applying both designed ddPCR assays for longitudinal follow-up (up to 500 days) of real-life DLBCL (n = 13) or B-ALL (n = 4) patients receiving either axi-cel (n = 10) or tisa-cel CAR-T cells (n = 7) (Fig. 5). For all of the patients, ddPCR was able to detect early expansion of CAR T cells, with a perfect match between ddPCR and flow cytometry. Similar kinetics, with early loss of CAR T cells after peak expansion, were detected by flow cytometry. However, in the vast majority of patients (16 out 17), ddPCR detects the persistence of circulating CAR T cells for up to 450 days (UPN #11).

Remarkably, in two B-ALL patients (UPN #14, Phi+ and #17) receiving tisa-cel, we noted in flow cytometry analysis that CD19+ leukemic cells reappear in the PB after being undetectable, whereas tisa-cel CAR T cells were always detectable by ddPCR.

## Discussion

The use of CD19 CAR T cells has been successful for treating R/R B cell malignancies such as ALL [18] or DLBCL, leading to several drug approvals by regulatory agencies, and this approach will be approved for other B-malignancies such as MM or CLL, and in the near future for AML and solid tumors in the long term. This success has underlined the need to precisely monitor CAR-expressing T cells.

Indeed, the administration of CAR T cells in clinical trials or in routine clinical practice has shown that early expansion and long-term persistence are predictive elements of the therapeutic response [19]. Longitudinal quantification of CAR T cells can inform CAR T cell engraftment and provide indirect information on adverse events such as cytokine release syndrome (CRS) and immune effector cell-associated neurotoxicity (iCANS) [9].

Moreover, in the case of relapse after CAR T cell infusion, it may be helpful to understand whether this relapse is caused by the loss of antigen by leukemic cells or other mechanisms independent of the presence or persistence of CAR T cells. This is well illustrated in our study, where 2 patients had detectable tisa-cel CAR T cells in the long term, while B cell numbers increased in the PB. Overall, it is now well established that direct monitoring of CAR T cells will help clinicians optimize and adapt treatment.

Today, there are different ways to quantify CAR T cells by flow cytometry using specific biotinylated targets or anti-scFv antibodies [20]. Because this method discriminates T cell subpopulations within circulating gene-modified T cells, its sensitivity remains limited. One way to increase sensitivity is to move toward the minimal residual disease (MRD) flow procedure by analyzing a large number of PB cells [21].

Other more sensitive molecular methods are available, taking advantage of the presence of additional nucleotide sequences integrated within the host genome of CAR T cells. LTR PCR is a well-proven and robust method that is used for routine HIV virus quantification and can be used for CAR T cell quantification by the presence of the LTR sequence promoter in lentiviral constructs [22]. However, in all the lentiviral backbones used for CAR constructs, the LTRs are deleted (self-inactivating (SIN) vector) to ensure safety and prevent self-replication. Such modifications may alter PCR primer annealing. Moreover, LTR PCR primers may cross-react with residual integrated homologous off-target sequences originating from previous infections.

Other approaches that target T cell signaling sequences in quantitative PCR assays, real-time (qPCR) [16] or endpoint PCR (dPCR) [23–25] are suitable for CAR T cell quantification. Here, we designed our own ddPCR assays based on 2 experimental CARs with known sequences, which were validated and tested in murine experiments. We demonstrated that our ddPCR assays also matched axi-cel and tisa-cel and allowed patient monitoring in the early or late stage of their treatment. While our ddPCR targets the T cell receptor junction sequence, it can easily be applied to a large panel of CAR T cells, such as brexu-cel (same construct as axi-cel), liso-cel and ide-cel, which carry the 4.1BB/CD3z junction. Moreover, ddPCR offers several advantages, as it does not need a reference standard curve, allows direct absolute quantification, is less sensitive to potential PCR inhibitors and is more accurate [17]. We noted that 28BBz is as sensitive as flow cytometry and less sensitive than 28z PCR. Analyzing a larger number of droplets or partitions may help to increase the sensitivity of the 28BBz PCR.

Another aspect is that ex vivo CAR T cell products need to meet quality control standards after manufacturing, such as vector copy number per cell, to meet regulatory requirements before injection [26, 27]. Using our own design ddPCR assay, quantification of VCN in the axi-cel medical product, previously quantified by the manufacturer, allowed us to validate the assay. Importantly, in preparation for a future phase I clinical trial, we were able to show that our IL-1RAP CAR T cells, produced at an MOI of 2 via either a research (Beads CD3/CD28+ IL-2) or GMP-like (soluble CD3/CD28+ IL-7/IL-15, in the cliniMACS prodigy device) protocol process, meet the regulatory requirements under a VCN threshold of 5 copies/transduced cell.

## Conclusion

Overall, we designed 2 ddPCR assays targeting the T signaling fusion area used in almost all CARs, which can be applied to experimental or approved (axi-cel or tisa-cel) CAR T cell quality control analysis and patient monitoring. The 28 BBz ddPCR assay will be elevated to the GMP level for application in quality control of our in-house IL-1RAP CAR T cell academic production process before being evaluated in an upcoming phase I clinical trial.

## Supplementary Information

**Additional file 1.** Supplementary data, Haderbache et al, qPCR, ddPCR and cytometry sensitivity and reproducibility

## Data Availability

All raw data are available on demand.

## Abbreviations

CAR: Chimeric antigen receptor
Tisa-cel: Tisagenlecleucel
Axi-cel: Axicabtagene-ciloleucel
Brexu-cel: Brexucabtagene autoleucel
Ide-cel: Idecabtagene vicleucel
FC: Flow cytometry
IL-1RAP: Interleukine 1 receptor accessory protein
CS1: SLAM family member 7, CD319
CD3z: CD3 zeta
VCN: Virus copy number
ddPCR: Digital droplet PCR
R/R: Refractory/relapsed
ALL: Acute lymphoblastic leukemia
DLBCL: Diffuse large B cell lymphoma
CLL: Chronic lymphoid leukemia
MM: Multiple myeloma
AML: Acute myeloid leukemia
CRS: Cytokine release syndrome
ICANS: Immune effector cell-associated neurotoxicity syndrome
BCMA: B-cell maturation antigen
qPCR: Real time quantitative real-time PCR
scFv: Single Chain Fragment Variable
gDNA: Genomic DNA
GMP: Good manufacturing production
ATMPs: Advanced therapy medical products
AUC: Area under the curve
MOI: Multiplicity of infection
UPN: Unique Personal Number
LTR: Long terminal repeat
CV: Coefficient of variation
SD: Standard deviation
