# Supplementary material for: Droplet digital PCR allows vector copy number assessment and monitoring of experimental CAR T cells in murine xenograft models or approved CD19 CAR T cell-treated patients
Source: J Transl Med. 2021 Jun 21;19:265. doi: 10.1186/s12967-021-02925-z (PMC8215786; doi:10.1186/s12967-021-02925-z)
Supplement: Supplementary file 1 — Additional file 1. Supplementary data, Haderbache et al, qPCR, ddPCR and cytometry sensitivity and reproducibility [file 12967_2021_2925_MOESM1_ESM.docx]

**Additional file**

**Droplet digital PCR allows vector copy number assessment and monitoring of experimental CAR T cells in murine xenograft models or approved CD19 CAR T cell-treated patients**

Rafik Haderbache et al,

**Table S1*.* 28z and 28BBz qPCR sensitivity and reproducibility.**

The mean, standard deviation (SD), and coefficient of variation (CV) for each DNA plasmid copy number dilution or for each gDNA cell dilution were calculated for both 28Z and 28BBz qPCR (n=4).

| **qPCR** | **Copies** | | **10e6** | **10e5** | **10e4** | **10e3** | **10e2** | **10e1** | **10e0** |  |  |
| --- | --- | --- | --- | --- | --- | --- | --- | --- | --- | --- | --- |
|  | **28z** | **Ct** | 20,38 | 23,78 | 27,29 | 30,99 | 33,45 | 34,41 | 34,53 |  |  |
|  |  | **SD** | 0,18 | 0,22 | 0,21 | 0,19 | 0,25 | 0,28 | 0,34 |  |  |
|  |  | **CV** | 0,45% | 0,34% | 0,18% | 0,55% | 0,47% | 0,25% | 0,45% |  |  |
|  | **28BBz** | **Ct** | 19,31 | 22,58 | 26 | 29,49 | 32,82 | 36,22 | 39,68 |  |  |
|  |  | **SD** | 0,11 | 0,08 | 0,1 | 0,11 | 0,25 | 0,26 | 0,67 |  |  |
|  |  | **CV** | 0,57% | 0,37% | 0,38% | 0,37% | 0,77% | 0,72% | 1,69% |  |  |
|  | **Cell dilutions** | | **undiluted** | **5.10e1** | **2.5.10e1** | **10e-1** | **10e-2** | **10e-3** | **10e-4** | **10e-5** | **10e-6** |
|  | **28z** | **Ct mean** | 25,68 | 27,85 | 29 | 29,76 | 33,65 | 36,88 | 36,72 | 38,58 | 37,48 |
|  |  | **SD** | 0,23 | 0,12 | 0,18 | 0,22 | 0,18 | 0,12 | 0,39 | 0,97 | 0,63 |
|  |  | **CV** | 0,90% | 0,43% | 0,63% | 0,75% | 0,52% | 0,33% | 1,07% | 2,52% | 1,67% |
|  | **28BBz** | **Ct mean** | 24,99 | 27,22 | 28,27 | 30,61 | 35,46 | 41,35 | - | - | - |
|  |  | **SD** | 0,29 | 0,06 | 0,1 | 0,31 | 0,48 | 1,65 | - | - | - |
|  |  | **CV** | 1,18% | 0,24% | 0,36% | 1,02% | 1,37% | 3,99% | - | - | - |

**Table S2*.* 28z and 28BBz ddPCR sensitivity and reproducibility.**

The mean, standard deviation (SD), and coefficient of variation (CV) for each DNA plasmid copy number dilution or for each gDNA cell dilution were calculated for both 28Z and 28BBz ddPCR (n=4).

| **ddPCR** | **Copies** | | **10e6** | **10e5** | **10e4** | **10e3** | **10e2** | **10e1** | **10e0** |  |  |
| --- | --- | --- | --- | --- | --- | --- | --- | --- | --- | --- | --- |
|  | **28z** | **Copies/µL** | 106 | 2278,9 | 374,7 | 38,42 | 3,93 | 1 | 0,7 |  |  |
|  |  | **SD** | 0 | 175,87 | 58,87 | 10,55 | 0,55 | 0,04 | 0,46 |  |  |
|  |  | **CV** | 0,00% | 7,72% | 15,71% | 27,45% | 13,93% | 4,05% | 66,12% |  |  |
|  | **28BBz** | **Copies/µL** | 2606,2 | 131,06 | 10,12 | 0,48 | 0,1 | 0 | 0,03 |  |  |
|  |  | **SD** | 54,82 | 14,09 | 2,47 | 0,11 | 0,1 | 0 | 0,06 |  |  |
|  |  | **CV** | 2,10% | 10,75% | 24,42% | 23,39% | 99,65% | - | 173,21% |  |  |
|  | **Cell dilutions** | | **undiluted** | **5.10e1** | **2.5.10e1** | **10e-1** | **10e-2** | **10e-3** | **10e-4** | **10e-5** | **10e-6** |
|  | **28z** | **Copies/µL** | 106,27 | 69,56 | 30,07 | 10,28 | 1,02 | 0,2 | 0,08 | 0,17 | 0,15 |
|  |  | **SD** | 5,82 | 1,28 | 1,42 | 1,01 | 0,44 | 0,07 | 0,15 | 0,05 | 0,11 |
|  |  | **CV** | 5,48% | 1,85% | 4,71% | 9,86% | 42,90% | 34,04% | 200,00% | 27,01% | 75,60% |
|  | **28BBz** | **Copies/µL** | 159,24 | 79,12 | 40,38 | 16,44 | 1,82 | 0,06 | - | - | - |
|  |  | **SD** | 0,94 | 2,02 | 3,46 | 2,12 | 0,3 | 0,07 | - | - | - |
|  |  | **CV** | 0,59% | 2,55% | 8,57% | 12,91% | 16,64% | 115,48% | - | - | - |

**Table S3*. Flow cytometry* sensitivity and reproducibility for CS1 or IL-1RAP CAR T cells.** Mean, standard deviation (SD), and coefficient of variation (CV) for dilution of each CEM transduced cell into untransduced CEM cells. (n=4). Myc staining was performed using an anti-myc antibody, and IL-1RAP staining was performed using a biotinylated recombinant protein and an anti-biotin *antibody.* Theoretical % is provided for the undiluted cell population and calculated according to the dilution factor.

| **FC** | **Cell dilutions** | | | **undiluted** | **5.10e1** | **2.5.10e1** | **10e-1** | **10e-2** | **10e-3** | **10e-4** | **10e-5** | **10e-6** |
| --- | --- | --- | --- | --- | --- | --- | --- | --- | --- | --- | --- | --- |
|  | **CS1 (28z)** | **Myc staining** | *Theoretical* | *98%* | *49%* | *24,5%* | *9,8%* | *0,98%* | *0,098%* | *0,0098%* | *0,00098%* | *0,000098%* |
|  |  |  | **Mean** | 94,80% | 46,58% | 23,44% | 9,30% | 0,97% | 0,12% | 0,05% | 0,11% | 0,04% |
|  |  |  | **SD** | 0,48% | 1,74% | 2,11% | 0,58% | 0,08% | 0,02% | 0,02% | 0,08% | 0,01% |
|  |  |  | **CV** | 1% | 4% | 9% | 6% | 9% | 16% | 38% | 70% | 34% |
|  | **IL-1RAP (28BBz)** | **CD19 staining** | *Theoretical* | *96%* | *48%* | *24,0%* | *9,6%* | *0,96%* | *0,096%* | *0,0096%* | *0,00096%* | *0,000096%* |
|  |  |  | **Mean** | 93,67% | 45,18% | 22,42% | 8,57% | 1,01% | 0,18% | 0,17% | 0,13% | 0,18% |
|  |  |  | **SD** | 2,71% | 3,57% | 1,93% | 1,61% | 0,13% | 0,03% | 0,08% | 0,08% | 0,19% |
|  |  |  | **CV** | 3% | 8% | 9% | 19% | 12% | 18% | 45% | 63% | 104% |
